# Skin Lesion Analysis for Melanoma Detection Using the Novel Deep Learning Model Fuzzy GC-SCNN

**DOI:** 10.3390/healthcare10050962

**Published:** 2022-05-23

**Authors:** Usharani Bhimavarapu, Gopi Battineni

**Affiliations:** 1 School of Competitive Coding, Koneru Lakshmaiah Education Foundation, Vaddeswaram, Vijayawada 522502, India; ushafdp1122@gmail.com; 2 Clinical Research Centre, School of Medicinal and Health Products Sciences, University of Camerino, 62032 Camerino, Italy

**Keywords:** fuzzy logic, GrabCut, convolution neural network, support vector machine, skin lesion

## Abstract

Melanoma is easily detectable by visual examination since it occurs on the skin’s surface. In melanomas, which are the most severe types of skin cancer, the cells that make melanin are affected. However, the lack of expert opinion increases the processing time and cost of computer-aided skin cancer detection. As such, we aimed to incorporate deep learning algorithms to conduct automatic melanoma detection from dermoscopic images. The fuzzy-based GrabCut-stacked convolutional neural networks (GC-SCNN) model was applied for image training. The image features extraction and lesion classification were performed on different publicly available datasets. The fuzzy GC-SCNN coupled with the support vector machines (SVM) produced 99.75% classification accuracy and 100% sensitivity and specificity, respectively. Additionally, model performance was compared with existing techniques and outcomes suggesting the proposed model could detect and classify the lesion segments with higher accuracy and lower processing time than other techniques.

## 1. Introduction

The skin’s vital role is to regulate body temperature as well as to protect against infections and injuries. Melanoma is a malignant growth of skin cells that typically develops on body parts that receive little or no sun exposure [1]. The number of skin cancer cases across the globe is reported to be around 5.4 million every year [2]. Several studies report an increase in the number of skin cancer cases in the United States from 95,360 in 2017 to 207,390 in 2021 [3,4].

Early detection and prevention of skin cancer reduce mortality rates [5]. The diagnosis of skin cancer is dependent on dermatoscopic training and experience. The patient’s clinical information is needed to screen the skin lesion due to the need to visualize morphological features not visible to the naked eye because of similar pixels and textures [6,7,8]. Dermatologists diagnose skin cancer based on conventional approaches such as color, diameter, and asymmetry. In comparison to conventional models, imaging technology allows more accurate and manual inspection of images while reducing time consumption and costs [9,10].

Each skin lesion has its shape, size, and border. Due to their intrinsic naivety, locality, and lack of adaptability, low-level hand-crafted features used by traditional methods and machine learning (ML) methods have limited discriminative properties. The existing literature highlighted the automatic detection of skin lesions by different ML models including gradient boosting [11], support vector machine (SVM) [12], and Quadtree [13]. SVM is used to classify the features extracted from the grey level co-occurrence matrix [14]. In [15], K-Nearest Neighbor (KNN) with a Gaussian filter is used to extract the region of interest (ROI) which is classified using SVM [15].

In the framework of medical image analysis, deep learning (DL) automates systems to detect, classify, and diagnose several diseases. These DL models are very effective for large sample datasets and, especially, they have become more viable for skin image analysis [16]. Some studies have compared the performance of DL models in the detection of skin lesions in several categories [17]. It is highlighted from the reports of [18,19] that the convolution neural network (CNN) is better performed than dermatologists in the segmentation of skin lesions [19,20,21]. These studies involved feature extraction techniques from segmented images that enabled quick diagnosis.

Other models such as deep neural networks (DNN), CNN, long short-term memory (LSTM), and recurrent neural networks (RNN) also help to detect malignant skin cells [22,23]. It is highlighted that CNN helps to detect dangerous skin cells from dermoscopy images which were found to be difficult to screen for nonmelanocytic and non-pigmented lesions [22]. In [23], a stacked CNN model with improved loss function was proposed to detect skin lesions from given datasets, and 94.8–98.4% classification accuracy was reported. The main drawbacks of previous approaches are that visual characteristics of skin lesion images contain inhomogeneous features and fuzzy boundaries, and the processing time.

Therefore, in this paper, we proposed an approach called fuzzy-based GrabCut-stacked convolutional neural networks (GC-SCNN) model with enhanced loss function in support vector machines (SVM). Additionally, we test the accuracy of the generated model and compare the outcomes of the enhanced Fuzzy GC-SCNN with existing techniques in lesion classification. Furthermore, this study aimed to understand the model’s effectiveness in the detection and classification of the lesion segments with better accuracy and lower processing time than other models.

## 2. Methods

### 2.1. Dataset

Various datasets of skin images were used including PH2 (http://www.fc.up.pt/addi/, accessed on 18 March 2022), and International Skin Imaging Collaboration (ISIC) 2018–2019 archives (http://isic-archive.com, accessed on 18 March 2022) for skin melanoma detection. There are 10,015 training images and 1512 test images in ISIC 2018, including lesion categories of melanoma, melanocytic nevus, basal cell carcinoma, actinic keratosis, benign keratosis, dermatofibroma, and vascular regions. The ISIC 2019 dataset contains 25,531 training images and 8238 test images divided into nine categories, including melanoma, melanocytic nevus, basal cell carcinoma, actinic keratosis, and benign keratosis, dermatofibroma, vascular regions, and an unknown class. PH2 datasets aimed at melanoma diagnosis and ISIC datasets are biased towards melanocytic lesions. They both focus on melanocytic lesions and disregarded the non-melanocytic lesions. The images available in the datasets are clinical images of skin images but not the dermoscopic images, so there is a mismatch between the available training images and the real-life data, which deviates the automated diagnostic system’s performance and builds a classifier for multiple skin diseases that is more challenging. 

HAM10000 (Human Against Machine) serves as a benchmark dataset for comparing humans and machines. This dataset consists of 10,015 dermatoscopic images of pigmented skin lesions with seven different categories: Actinic Keratoses and Intraepithelial Carcinoma (AKIEC), Basel Cell Carcinoma (BCC), Benign Keratosis-like Lesions (BKL), Dermatofibroma (DF), Melanoma (MEL), Melanocytic Nevi (NV), and Vascular Lesions (VASC) [24]. We decomposed the image dataset into an 80:20 ratio where 80% was used for training and 20% for testing.

### 2.2. Data Preprocessing

**Figure 1:**
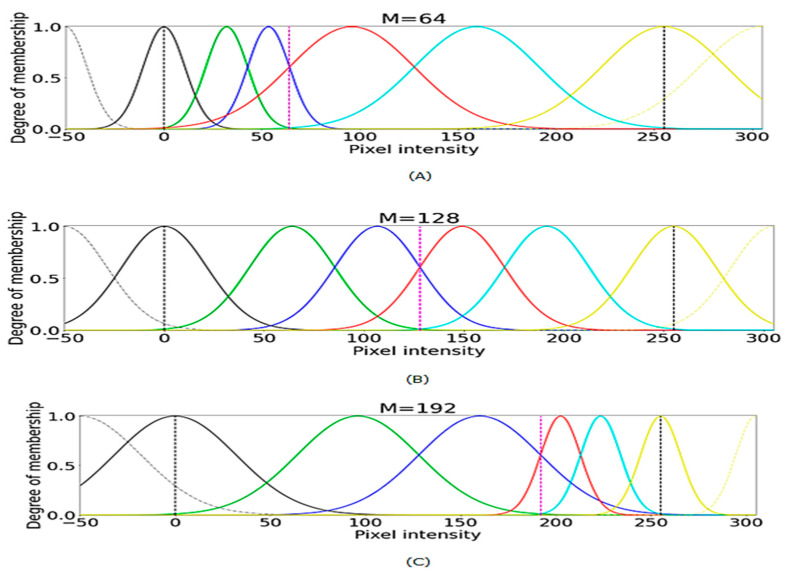
The degree of membership of three different channels with (**A**) M = 64, (**B**) M = 128, and (**C**) M = 192.

**Figure 2:**
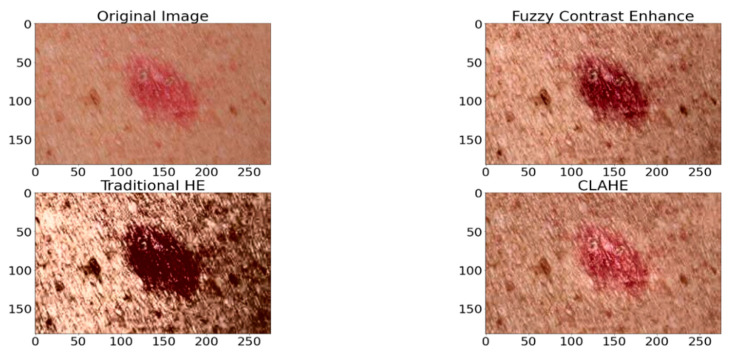
Preprocessed Image.

The original dermoscopy image sizes varied from 540 × 576 to 2016 × 3024. We applied the image resizing and maintained the uniform image size of 256 × 256. The morphological filtering and marker concepts were adopted to highlight the melanoma region and skin hair removal. These morphological filters are used for image sharpening. Erosion and dilation are the two basic morphological operators, where dilation selects the brightest value near the structuring element. The membership functions of dermoscopic images with different channels can be observed in Figure 1. The preprocessing of the dermoscopy images (Refer to Figure 2) for enhancement and detection of the lesion boundaries was conducted as mentioned below.

❖ The pixels of the skin lesion domain are taken to a fuzzy domain. Let *M* be an image of *p* × *q*, and *M* (*p*,*q*) represent the intensity of the skin lesion image pixels that must be mapped to the fuzzy characteristic plane. It can be expressed as follows $M\left(p,q\right)=\overset{m}{\underset{p=1}{\sum }}\overset{n}{\underset{q=1}{\sum }}\frac{\mu M\left(i,j\right)}{M\left(i,j\right)}$, *p* = 1, …, *m* and *q* = 1, …, *n*; where $\frac{\mu M\left(i,j\right)}{M\left(i,j\right)}$ represent the pixels and *µM* (*p*, *q*) is the intensity level degree of the image ranging from zero to one.
❖ Assign the fuzzy plane pixels to the logarithmic function to map to the fuzzy domain f (M (*p*, *q*)) = $\log_{2}$((1 + $\frac{M\left(p,q\right)-M_{\max}}{M_{\max\;}+M_{\min}}$); where* M*_max_ and *M*_min_ are the maximum and minimum intensity of the skin lesion image pixels.
❖ To enhance the portions of the skin lesion images, transform the image using the trigonometric series with fuzzy principles as mentioned f (T (*p*, *q*)) = T (*p*, *q*) + f (*M* (*p*, *q*))^2^ where 0 ≤ f (*M* (*p*, *q*)) ≤ 0.5; where T (*p*, *q*) =$\;\frac{\tan\left(a\right)}{4}+\frac{\cos\left(a\right)}{3}$ and a = π (f (*M* (*p*, *q*) − 0.5) + 1.
❖ The defuzzification can be expressed as D = *M*_min_ + ((*M*_max_ − *M*_min_) × 2 ^T(*p*, *q*)^ − 1)
❖ Later, enhance the image quality by skin lesion image channel-wise.

### 2.3. Image Segmentation

**Figure 3:**
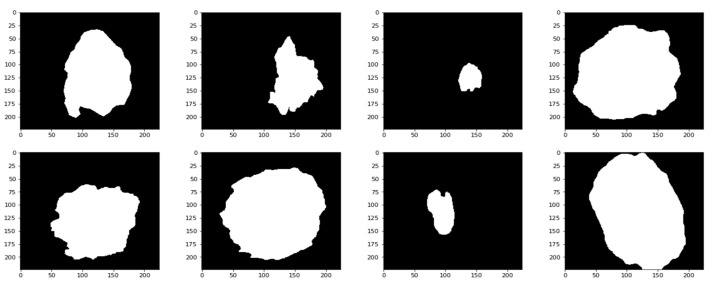
Segmented lesions.

In this work, the segmentation phase has done by the GrabCut (GC) segmentation that is used to segment the fuzzy preprocessed image. Figure 3 shows the results of segmenting the data and identifying the necessary areas. 

Let the color image be represented with x and the array of pixels represented as the y = (y_1_, y_2_, …, y_n_) where each zi = (R_i_, G_i_, B_i_), i ∈ [1, …, n]. During the segmentation, the label of the pixels is represented as the $\beta =(\beta_{1},\;\beta_{2},\;\;\ldots ,\beta_{n})\;\mathrm{where}\;\beta_{i}\in \;${0,1}. The trimap with a semiautomated direction can be applied to three regions called the background, foreground, and the uncertain pixels and they can be represented as Z_B_, Z_F_, and Z_U_. The covariance of the gaussian mixture model of n elements is determined using the background pixels and the foreground pixels.

α = {Π(β,k),μ(β,k),Σ(β,k)}; where Π, μ,Σ are the weight, mean and covariance matrices and k = {$k_{1},\;\;k_{2},\;\;\ldots ,k_{n}$} where $k_{i}\in \;${1, …, n} for the elements of the gaussian mixture model of the pixels $y_{i}$.

The function for the segmentation can be expressed as F(β,k,α,y) = P(β,k,α,y) + R (β,y); where *P *represents the probability distribution Z of the gaussian mixture model and R represents the regularizing of the segregated regions concerning the color and the neighborhood pixels and R assumes the neighborhood E over the pixels

$$P\left(\beta ,k,\alpha ,y\right)\;=\sum_{m}-log\;z\left(y_{m}|\beta_{m},k_{m},\alpha \right)-log\;\Pi \left(\beta_{m},k_{m}\right)$$

$$R\left(\beta ,y\right)\;=\vartheta \sum_{\left\{i,j\right\}\in E}\left[\beta_{i}\neq \beta_{j}\right]exp\left(\theta \|y_{i}-y_{j}\|{}^{2}\right)$$

### 2.4. Feature Extraction

After performing the segmentation, we applied the stacked CNN technique to extract the corresponding features in the segmented image. The proposed hybrid approach learns nonlinear discriminative features from the dermoscopy images at different levels. Algorithm 1 discusses the GC-SCNN Algorithm. CNN automatically learns the valuable features, and we integrated three modules, Inception-V3 [25], Xception [26], and VGG-19 [27]. In the first module, pre-trained Inception-V3, Xception, and VGG-19 models are tuned for dermoscopy images to extract features from the segmented image. The second model of the stacked CNN obtained six sub-models during the training of the CNN models. We stacked together all the sub-models and then applied the SVM classification to build a model to classify the lesions. The Algorithm 1 for GC-SCNN is written as
**Algorithm 1:** GC-SCNN.*Input:* Segmented Images *Output:* Skin cancer Classification results for k = 1 to length (segmented images) do for j = 1 to 3 do sub-model j. predicts (segmented image) end for final = concatenation (P_1_, P_2_, P_3_) end for assess the SoftMax classifier on the feature vector final stacked CNN = Train (final, label) classification of skin cancer images prediction = classification (stacked CNN, testset) return prediction

### 2.5. Lesion Classification

The SVM classifier takes the extracted features and classifies the lesion. First, the SVM calculates the feature score by using the linear mapping on feature vectors and uses this feature score to calculate the loss. The loss should be minimal to get better accuracy; we use an improved loss function [28] to calculate the weighted score for each pixel in the segmented lesion image. The Algorithm 2 for the enhanced SVM is described as
**Algorithm 2:** Enhanced SVM algorithm.Initialize the values in the training set Repeat for every i = 1 to N calculate the loss function for all values compare the extracted patches in the images end for Repeat for every score vector i- 1 to N Compute SVM with imputed labels argmax((w × xi) + b), i end for Evaluate for different weights and compute output.

Enhanced SVM reduces the number of neurons, leading to overfitting minimization, increasing accuracy, and reducing processing time. The enhanced loss function reduces the load of the segmented dermoscopy images fed to the enhanced SVM classifier, which reduces the processing time. The improved loss function in the existing SVM algorithm improves the performance in classifying the lesion segments depending on the intensity and the score vectors.

### 2.6. Experimental Framework

**Figure 4:**
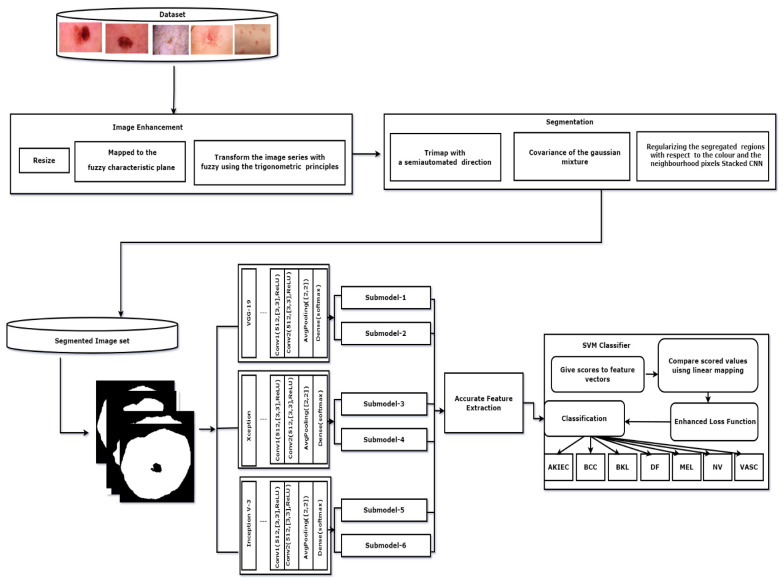
Block diagram of the proposed model.

The proposed methodology discusses the classification of skin lesions. First, we fed the input data for preprocessing using fuzzy logic to enhance the image and identify the lesion boundaries. We then applied the morphological operators to remove the hair on the skin. Then the images were sent for segmentation using the GrabCut technique. Later, the features were selected using the stacked CNN. Finally, to classify the lesions, we used the improved SVM classifier. The proposed experimental framework is illustrated in Figure 4.

### 2.7. Performance Metrics

The performance metrics can help measure the model presented in terms of the different parameters mentioned below. For instance,

❖ Accuracy measures the portion of the true results among the total number of the cases and is written as accuracy = $\;\frac{\mathrm{TP}\;+\;\mathrm{TN}}{\mathrm{TP}\;+\;\mathrm{TN}\;+\;\mathrm{FP}\;+\;\mathrm{FN}}$; Where FP-False positive, FN-False Negative, TP-True Positive, TN-True Negative
❖ Sensitivity is the portion of the positive outcomes among the actual positive and it is defined as sensitivity = $\frac{\mathrm{TP}}{\mathrm{TP}\;+\;\mathrm{FN}}$
❖ Specificity is defined as the portion of the true negative outcomes among the negative outcomes and it is written as specificity = $\;\frac{\mathrm{TN}}{\mathrm{TN}\;+\;\mathrm{FP}\;}$

## 3. Results

The performance of the stacked CNN frameworks is assessed in this section and compared to the performance of the existing models, and the dataset is decomposed into an 80:20 ratio. For simulation, the enhanced fuzzy-SCNN was implemented in Python with IDE Anaconda on the Intel Core i5 3.4 GHz processor.

Deep learning models require a robust set of hyperparameters. Hyperparameter tuning enhances deep learning performance. Various optimization techniques exist for hyperparameters and the manual search technique is one of them. A variety of hyperparameter combinations have been tested and the best model has been selected. Various hyperparameters were set for optimizers, learning rate, weight decay value, and dense layers. Hyperparameters in the network include the learning rate, optimizer, dense layers, and decay constant. A stacked CNN is tuned by varying its hyperparameters, as shown in Table 1. Optimizing algorithms affect both training speed and prediction accuracy. The popular optimizer algorithms in deep learning are the Root Mean Square Propagation (RMSProp), adaptive Moment Optimization (Adam), Stochastic gradient descent (SGD), and Adaptive Gradient (AdaGrad), Adadelta.

**Figure 5:**
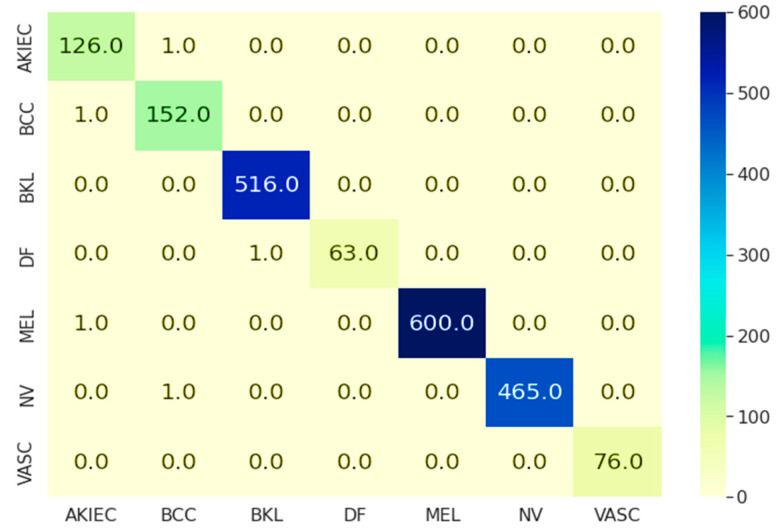
Confusion matrix.

Different hyperparameters were varied for each optimizer and accuracy was compared. With both Adam and RMS prop tuned with various hyperparameters, the Adam optimizer had the best performance, followed by AdaGrad and Adadelta. We used two values of the learning rates, 0.01, 0.001, weight decay constants of 0.01 and 0.001, and dense layers of 4 and 5, which required a batch size of 64. With all of these settings, the performance was highest with the least amount of computing resources. With batch size 32, optimizer ADAM, dense layers 4 with learning rate and weight decay constants of 0.0001 and 0.0001, we achieved low loss. These hyperparameters were used to classify skin lesions. The model training included seven categorical skin lesions and the model performance was assessed with the confusion matrix outcome shown in Figure 5. AKIEC, BCC, and BKL lesion classes were predicted with 99.21%, 99.34%, and 100% accuracy, respectively. In contrast, DF, MEL, NV, and VASC had 98.437%, 99.83%, 99.78%, and 100% prediction accuracy. The overall model accuracy was reported as 99.75%. Other metrics such as sensitivity (true positive rate) and specificity (true negative rate) were achieved at 100% which is higher than other previous studies.

Table 2 presents the comparison of different existing models with the GC-SCNN over the test dataset. In Table 3 and Table 4, we compare the proposed model to the state-of-the-art approaches to the ISIC2018 and ISIC2019 datasets. Based on the accuracy figures of 99.78% (for ISIC 2018) and 99.81% (for ISIC 2019), as a result, the proposed model outperforms by 1% and 2.5%.

## 4. Discussion

An automatic skin lesion detection method based on fuzzy GC-SCNN is presented in this paper. For boundary detection and segmentation, we used fuzzy logic, stacked CNNs for feature extraction, and enhanced SVMs for lesion segmentation. At different stages of lesion classification, the enhanced fuzzy GC-SCNN with SVM was compared with existing techniques. It was proven that the proposed model was more accurate and faster at classifying the lesion segments than other models, and produces very few false positives and false negatives.

A skin lesion’s detection and classification performance are typically affected by discriminant feature selection [48]. Existing literature on this topic does not elaborate on image processing steps and does not address the uncertainty of detecting lesion boundaries. For example, in [49], the authors proposed the use of orthogonal matching with a fixed wavelet grid network to enhance, segment, and classify demographic images and obtained an accuracy of 91.82%. By combining SVM, SMOTE, and ensemble classifiers in combination with extracting color texture features from dermoscopy images, 93.83% accuracy was achieved [50]. It was also possible to extract color, texture, and SVM features by using the Gray Level Co-Occurring Matrix (GLCM) technique [51].

Some studies have achieved improved accuracy in skin malignant cell prediction through threshold-based segmentation, ABCD feature extraction, and multiscale lesion-biased techniques [52,53,54]. A CNN model comprised of multiple tracks was developed to resolve the issue of skin lesion classification. The model achieved 85.8% and 79.15% accuracy over five and ten classes respectively [55,56]. In contrast, ensemble-based deep learning demonstrated improved performance in skin lesion classification and reported approximately 90% accuracy [57,58]. Despite this, all of the above-mentioned studies applied a single model, which can affect the accuracy of the model. By stacking different models, we could improve the accuracy.

Based on the Delaunay triangulation, a study with two parallel processes was able to detect skin lesions [59]. The backpropagation multilayer neural network was used to detect and classify melanoma using 3D color texture features from dermoscopy images [60]. On ImageNet datasets, the transfer learning approaches with the CNN model produced 88.33% of accuracy thanks to pre-trained models like Resnet-101, BASNet large, and Google Net [61]. All of these approaches have the disadvantage that in medical diagnosis they require prolonged real-time analysis. Our method of detecting lesion boundaries via fuzzy image processing overcame these limitations.

Additionally, our study is in line with [62] as the authors applied transfer learning to train the model with the HAM1000 dataset. They implemented Resnet50 models with no data preprocessing and manual feature selection which resulted in a significant decrease in the model accuracy and high processing time. The enhanced fuzzy-SCNN with SVM improved the classification accuracy by reducing the loss and achieved 99.75% accuracy. By minimizing the overfitting of training datasets in the SVM classifier, we improved the classification performance by using the same data set for the newly developed and existing models. A modified loss function improved lesion classification by reducing processing time by 25–35 milli seconds and increasing accuracy by 2–5%.

Identifying and classifying seven significant lesions in dermoscopy images was possible with the proposed solution. Although our solution produced the best possible accuracy, we have focused on only a limited set of lesions while neglecting minute lesions. Future work will involve improving the feature extraction techniques with latent factor analysis to detect negligible minute lesions [63,64]. Incorporating more lesion types with lower noise by neural network architecture can enhance the model’s significance.

## 5. Conclusions

Human beings are protected by their skin against environmental pollution, but the adverse effects of ultraviolet radiation increase the risk of melanoma. We propose a deep learning framework to segment, detect, and classify skin lesions in dermoscopy images for melanoma detection. Based on the publicly available dataset HAM10000, which consists of seven lesion categories, we evaluated the proposed framework. Our model outperformed the existing models in terms of performance. As a result of the current study, the uncertainties in boundary detection were removed, reducing the loss and the processing time. We calculated the prediction time of the proposed model and lesion detection takes 2.513 ms. In conclusion, the results suggest that the proposed model is computationally efficient.

## Figures and Tables

**Table 1:** Different hyperparameter tuning effects.

Batch Size	Optimizer	Dense	Learning Rate	Weight Decay Values	Epoch	Loss	Processing Time (ms)
32	RMSProp	4	0.0001	0.0001	50	6.50	4
	RMSProp	4	0.0001	0.001	100	6.56	5
	RMSProp	4	0.001	0.0001	50	6.25	4
	RMSProp	4	0.001	0.001	100	6.57	5
64	RMSProp	4	0.0001	0.0001	50	7.27	5
	RMSProp	4	0.0001	0.001	100	7.37	6
	RMSProp	4	0.001	0.0001	50	7.52	5
	RMSProp	4	0.001	0.001	100	7.79	7
32	RMSProp	5	0.0001	0.0001	50	8.46	6
	RMSProp	5	0.0001	0.001	100	8.63	7
	RMSProp	5	0.001	0.0001	50	8.21	6
	RMSProp	5	0.001	0.001	100	8.35	7
64	RMSProp	5	0.0001	0.0001	50	8.32	8
	RMSProp	5	0.0001	0.001	100	8.34	7
	RMSProp	5	0.001	0.0001	50	8.25	7
	RMSProp	5	0.001	0.001	100	8.31	7
32	ADAM	4	0.0001	0.0001	50	6.26	3
	ADAM	4	0.0001	0.001	100	6.28	4
	ADAM	4	0.001	0.0001	50	6.27	4
	ADAM	4	0.001	0.001	100	6.55	5
64	ADAM	4	0.0001	0.0001	50	7.04	4
	ADAM	4	0.0001	0.001	100	7.06	5
	ADAM	4	0.001	0.0001	50	7.26	4
	ADAM	4	0.001	0.001	100	7.27	6
32	ADAM	5	0.0001	0.0001	50	7.67	4
	ADAM	5	0.0001	0.001	100	7.63	5
	ADAM	5	0.001	0.0001	50	7.21	4
	ADAM	5	0.001	0.001	100	7.35	6
64	ADAM	5	0.0001	0.0001	50	8.02	4
	ADAM	5	0.0001	0.001	100	8.14	5
	ADAM	5	0.001	0.0001	50	8.05	5
	ADAM	5	0.001	0.001	100	8.10	6
32	AdaGrad	4	0.0001	0.0001	50	6.47	4
	AdaGrad	4	0.0001	0.001	100	6.74	4
	AdaGrad	4	0.001	0.0001	50	6.25	5
	AdaGrad	4	0.001	0.001	100	6.55	5
64	AdaGrad	4	0.0001	0.0001	50	7.14	5
	AdaGrad	4	0.0001	0.001	100	7.06	6
	AdaGrad	4	0.001	0.0001	50	7.16	6
	AdaGrad	4	0.001	0.001	100	7.29	7
32	AdaGrad	5	0.0001	0.0001	50	7.77	5
	AdaGrad	5	0.0001	0.001	100	7.61	6
	AdaGrad	5	0.001	0.0001	50	7.23	6
	AdaGrad	5	0.001	0.001	100	7.32	7
64	AdaGrad	5	0.0001	0.0001	50	8.06	5
	AdaGrad	5	0.0001	0.001	100	8.18	5
	AdaGrad	5	0.001	0.0001	50	8.09	6
	AdaGrad	5	0.001	0.001	100	8.11	7
32	Adadelta	4	0.0001	0.0001	50	6.69	4
	Adadelta	4	0.0001	0.001	100	6.56	5
	Adadelta	4	0.001	0.0001	50	6.28	4
	Adadelta	4	0.001	0.001	100	6.47	4
64	Adadelta	4	0.0001	0.0001	50	6.85	4
	Adadelta	4	0.0001	0.001	100	7.44	4
	Adadelta	4	0.001	0.0001	50	7.16	5
	Adadelta	4	0.001	0.001	100	7.26	5
32	Adadelta	5	0.0001	0.0001	50	7.67	6
	Adadelta	5	0.0001	0.001	100	7.77	6
	Adadelta	5	0.001	0.0001	50	7.73	5
	Adadelta	5	0.001	0.001	100	7.31	7
64	Adadelta	5	0.0001	0.0001	50	7.55	6
	Adadelta	5	0.0001	0.001	100	8.08	7
	Adadelta	5	0.001	0.0001	50	8.19	5
	Adadelta	5	0.001	0.001	100	8.12	6

**Table 2:** HAM10000 comparison of classification.

Classifier	Accuracy (%)	Sensitivity (%)	Specificity (%)
DCN transfer learning [29]	94.92	80.36	79.8
Mobile Net [30]	83.1	89	83
Kernel extreme learning machine [31]	90.67	90.20	89.43
DilatInceptV3 [32]	90.10	87	87
Proposed	99.75	100	100

**Table 3:** ISIC2018 comparison of classification.

Project	Accuracy (%)	Sensitivity (%)	Specificity (%)
Gessert et al. [33]	98.70	80.9	98.4
Ailin et al. [34]	98.20	89.5	98.1
Khan et al. [35]	89.80	89.7	94.5
Mohamed et al. [36]	92.70	72.42	97.14
Huang et al. [37]	85.80	69.04	95.92
Liu et al. [38]	92.54	71.47	92.72
Gu et al. [39]	91.4	83.74	93.24
Zhou et al. [40]	92.55	84.67	93.63
Gan et al. [41]	93.81	90.14	98.36
Proposed	99.78	100	100

**Table 4:** ISIC2019 comparison of classification.

Project	Accuracy (%)	Sensitivity (%)	Specificity (%)
Gessert et al. [33]	92.3	80.9	98.4
Ailin et al. [34]	91.5	89.5	98.1
Ahmed et al. [42]	94	89.7	94.5
Pacheco et al. [43]	92	72.42	97.14
Molina et al. [44]	97	69.04	95.92
Kaseem et al. [45]	94	71.47	92.72
Iqbla et al. [46]	90	83.74	93.24
Pulgarin et al. [47]	92	89.53	93.57
Proposed	99.51	100	100

## Data Availability

Not applicable.
